# CREATING DESIGN CONNECTIONS: ARTS-BASED METHODS AS A PATHWAY INTO TRAUMA-INFORMED DESIGN FOR OLDER HOMELESS ADULTS

**DOI:** 10.1093/geroni/igae098.0952

**Published:** 2024-12-31

**Authors:** Alison Grittner, Christine Walsh

**Affiliations:** Cape Breton University, Sydney, Nova Scotia, Canada; University of Calgary, Calgary, Alberta, Canada

## Abstract

Older adults who have experienced homelessness are more likely to live with trauma than their continually housed counterparts. Trauma-informed design (TID) recognizes that the built environment – defined as all physical elements that are human-made or curated to respond to human needs, desires, or purposes – impacts the physical, psychological, and emotional effects of trauma. TID seeks to positively shape this trauma-built environment connection through intentional design. While environmental gerontology has long understood the connection between aging well and the built environment, understanding trauma-informed design for aging adults is a new perspective. In this presentation, we explore the importance of collaborative and artful methodological strategies for understanding the TID needs of older adults with experiences of homelessness, generated by a secondary data analysis of 35 arts-based interviews with older adults (ages 50-71) living in four supportive housing sites in Calgary, Canada. We share our methods (photovoice and arts-based elicitation), examples of artful knowledge concerning the built environment created by research participants, and insights for translating art-based findings into design strategies, using our own research project as an exemplar. Overall, we demonstrate the possibilities of arts-based methods to understand, design, and create trauma-informed supportive housing for older adults with experiences of homelessness that fosters wellness, slows aging, and promotes healing from trauma.

